# Ethical Conflicts in Healthcare Chaplaincy: Results of an Exploratory Survey Among Protestant Chaplains in Switzerland, Germany, and Austria

**DOI:** 10.1007/s10943-022-01681-8

**Published:** 2022-11-23

**Authors:** Sebastian Farr, Traugott Roser, Michael Coors

**Affiliations:** 1 Institute of Social Ethics, Center for Ethics, University of Zurich, Zürich, Switzerland; 2 Seminar für Praktische Theologie und Religionspädagogik, University of Münster, Münster, Germany

**Keywords:** Healthcare chaplaincy, Pastoral care, Ethics, Ethics consultation, End-of-life care

## Abstract

The paper reports the results of an exploratory online survey among German, Austrian, and Swiss hospital chaplains (*n* = 158, response rate 17%) to identify the ethical conflicts they encounter in their work. Respondents indicated that questions surrounding end-of-life care are predominant among the conflicts faced. Chaplains get involved with these conflicts most often through the patients themselves or through nursing staff. Most encounters occur during pastoral care visits rather than in structured forms of ethics consultation such as clinical ethics committees. The results add to the ongoing discussion of chaplains as agents in ethics consultation within healthcare systems as well as their specific role and contribution.

## Introduction

Theoretical research has acknowledged that chaplains are confronted with a wide variety of moral conflicts, that they can be involved in the consultancy process with patients, family, or staff, and that they deal with these conflicts in both structured (i.e., in clinical ethics committees) or non-structured forms (i.e., during personal patient contact).

Within the German-speaking context, empirical research has been either conducted with small sample sizes taking a qualitative approach or has focused on a specific field of ethics conflict—especially end-of-life care (Clemm, 2015; Clemm et al., 2015; Moos et al., 2016). The survey we conducted therefore built upon these previous findings, but widened the field of view to gather broader data on the types of ethical conflicts chaplains encounter as well as team constellations and settings that lead to chaplains being involved. It thereby also leans upon analogous quantitative research from Australia and New Zealand that has surveyed chaplains in their respective healthcare systems in similar ways (Carey, 2012; Carey & Cohen, 2008; Carey et al., 2006).

We assume that the integration of chaplains in the care of patients not only provides spiritual support to patients, but also benefits the ethical discussion of care in both individual cases and on an institutional level. Even though healthcare and medical ethics have been established as secular academic disciplines and clinical ethics is widely performed by physicians, it is important to acknowledge that healthcare chaplains have been involved within these ethics deliberation processes from the beginning of structured forms in the German-speaking contexts (cf. Deutscher Evangelischer Krankenhausverband e.V. & Katholischer Krankenhausverband Deutschlands e.V., 1997). Chaplains therefore need to be considered as agents within the ethics network that is present in hospitals and as agents who take care of patients in their own way (Mandry et al., 2019).

Our research aims at providing a better understanding of the ways chaplains care for patients in terms of providing helpful resources in ethical conflicts as well as their specific position within the healthcare institution.

## Method

### Design and Sample

The survey was directed at Protestant hospital chaplains in Germany, German-speaking Switzerland, and Austria, who currently hold such a position—either part time or full time.

The design of the questionnaire was mainly informed by a survey conducted by Clemm et al., (2015; Clemm 2015) on the role of chaplains in end-of-life decision-making, as well as surveys of Lindsay Carey (2012; Carey et al., 2006) on healthcare chaplaincy in Australia and New Zealand, and a qualitative study on ethics in chaplaincy by Thorsten Moos et al. (2016)—all of them questioned similar groups and worked on related topics of research.

### Survey Instrument

The survey was conducted via an online questionnaire containing 54 items on demographic data, general information on the hospital at which the chaplains worked, types of ethical conflicts they engage, and information on their level of institutional engagement with ethics consultation.

For general data on demographic and workplace information, the questions were mainly single or multiple choice. The information provided on ethical conflicts, communication settings, and interactions was presented as relative frequencies indicated as “never,” “rarely,” “sometimes,” and “often.” This relative scale allowed for chaplains of different weekly working hours to give an indication of their engagement relative to one another.

The questions were asked in German. It took participants on average 9.7 min to complete the survey. Questions and answer options have been translated as close as possible for the purpose of this paper.

### Questionnaire Administration

The survey had been sent out via an email containing the link to the online survey to all chaplains through different channels for the different countries: In Germany, the board of the chaplaincy conference of the “Evangelische Kirche in Deutschland” (EKD) sent an email to all delegated members of each province church, who then proceeded to forward the link through their channels to all chaplains in their province. In German-speaking Switzerland, the board of the association of chaplains (Vereinigung der deutschschweizerischen evangelischen Spital-, Heim- und Klinikseelsorger und -seelsorgerinnen) emailed the link to all members, who fit the profile of participants. In Austria, the email was sent out by the coordination office for evangelical hospital chaplaincy by church officials. All forwarding parties were asked to review and approve the survey in advance to it being sent out.

The survey link in the email was accompanied by a brief introduction on the research goals of the survey. This explanation was also displayed to participants before starting the questionnaire together with an informed consent form on data processing.

The survey period lasted 31 days with a reminder for the survey being sent out after 18 days (June 25 – July 26, 2020).

### Quantitative Analysis

The dataset was exported from the online survey via the provided export tool. The statistical analysis was performed using the statistical programming language R (R Core Team, 2022) accompanied by RStudio (RStudio Team, 2020). All plots were created using the package ggplot2 (Wickham, 2016).

Testing of predetermined hypotheses was performed through correlation analysis for non-directional correlations between two variables and two-sample t tests. For visualization purposes, confidence intervals have been calculated and displayed in some plots.

For the purpose of analysis, individuals whose answers contained “NA” in individual questions were still considered, but taken out of the analysis of the particular question or connected hypothesis test. For significance testing, at least 2 *σ* needed to be reached for a result to be considered significant.

## Results

### Response Rate

The data on the response rate of the survey is based on feedback of the organizations that forwarded the survey instrument. However, some mentioned that chaplains could be registered with more than one email address in the corresponding database. Based on this feedback, the survey reached 935 individuals. Additionally, it cannot be ruled out that survey participants forwarded the link to colleagues. The main benefit of this approach was that it allowed the survey to reach a large number of chaplains easily while still obeying data protection regulations, since the associations or churches could not provide any database on personal contact information to the researchers.

During the 31 days of run time, 182 individuals completed the survey. Out of these, 158 indicated that they currently hold a position as hospital chaplain and are therefore evaluable for the purpose of this survey (*n* = 158; cf. Table 1 and 2). The response rate was estimated at 17%.

**Table 1:** Demographic information (*n* = 158)

*Gender*		
Female	97	(61.4%)
Male	59	(37.3%)
Prefer not to say	1	(0.6%)
*Age*		
30 – 39	10	(6.3%)
40 – 49	13	(8.2%)
50 – 59	91	(57.6%)
60 – 69	43	(27.2%)
*Religious affiliation*		
Ev. Luth./AB	76	(48.1%)
Ev. Ref./HB	26	(16.5%)
Ev. Uniert	42	(26.6%)
Rom.-Catholic	11	(7.0%)
Other	3	(1.9%)
*Training and qualification*		
Study of theology	134	(84.8%)
Theology lateral entry	6	(3.8%)
Social welfare work/deaconry	19	(12.0%)
Further studies: pastoral care	19	(12.0%)
Further training: pastoral care	101	(63.9%)
Further studies: ethics consultancy	10	(6.3%)
Further training: ethics consultancy	59	(37.3%)
Formal training/studies: medicine or nursing	13	(8.2%)
*Chaplaincy experience*		
Under 2 Years	16	(10.1%)
2 – 5 Years	32	(20.3%)
6 – 9 Years	28	(17.7%)
Over 9 Years	81	(51.3%)
*Country*		
Germany	116	(73.4%)
Switzerland	30	(19.0%
Austria	11	(7.0%)

**Table 2:** General data (*n* = 158)

*Sponsorship of position*		
Church	119	(75.3%)
Hospital	18	(11.4%)
Other	19	(12.0%)
*Type of hospital*		
University Hospital	45	(28.5%)
Hospital of maximum care	55	(34.8%)
Hospital of primary care	30	(19.0%)
Specialized Hospital	27	(17.0%)
*Duration in specific hospital*		
Under 2 Years	26	(16.5%)
2 – 5 Years	41	(26.0%)
6 – 9 Years	23	(14.5%)
Over 9 Years	65	(41.1%)
*Structured forms of ethics deliberation in hospital*		
Structure(s) established	142	(89.9%)
Specific ethics personal employed	63	(43.8%)
Involvement of responding chaplain	96	(66.7%)

This response rate was lower than in other recent surveys of hospital chaplains (e.g., Clemm et al., 2015 indicated a response rate of 59%). A key difference between the two surveys is that Clemm et al. obtained an address list of chaplains being involved with the “Workgroup Spiritual Support” of the German Society for Palliative Medicine (Arbeitskreis Spirituelle Begleitung der Deutschen Gesellschaft für Palliativmedizin)—therefore directly contacting particularly well-organized and involved chaplains.

Additionally, the COVID-19 pandemic might have led to a higher workload for hospital chaplains and therefore less time resources to participate in surveys. Also, further survey requests had been sent out to chaplains during the COVID-19 pandemic (the European Research Institute for Chaplaincy in Healthcare conducted a survey on COVID-19 effects on hospital chaplaincy in May and June, cf. Vandenhoeck, 2021).

### Areas of Ethical Conflicts

Earlier studies have often focused largely on chaplains’ involvement with ethical decision-making in the context of end-of-life care (Carey, 2012; Carey et al., 2006; Clemm, 2015; Clemm et al., 2015). Other surveys have indicated a larger variety of fields of conflict from smaller sample sizes with a more qualitative research approach (Moos et al., 2016, pp. 40–46). As stated above, one major aim of this survey therefore was to obtain information on the variety of fields of ethical conflicts chaplains engage with.

**Fig. 1:**
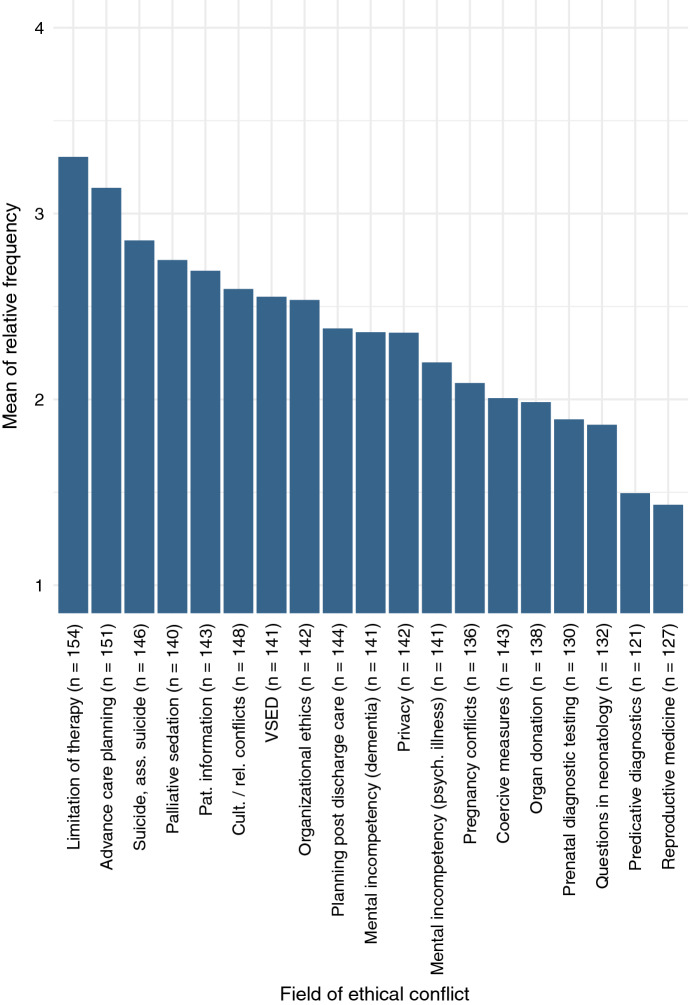
Fields of ethical conflict responding chaplains had contact with. Scale: 1 = never, 2 = rarely, 3 = sometimes, 4 = often

In this study, chaplains were asked to indicate the relative frequency on a scale of 1–4 (“never,” “rarely,” “sometimes,” and “often”) for 19 different fields of ethical conflict as shown in Fig. 1.

The results in Fig. 1 show that the frequency of occurrences varies greatly between different fields of conflict. Overall, it can be said that ethical questions surrounding the end of life appear most often in chaplains’ praxes.

**Fig. 2:**
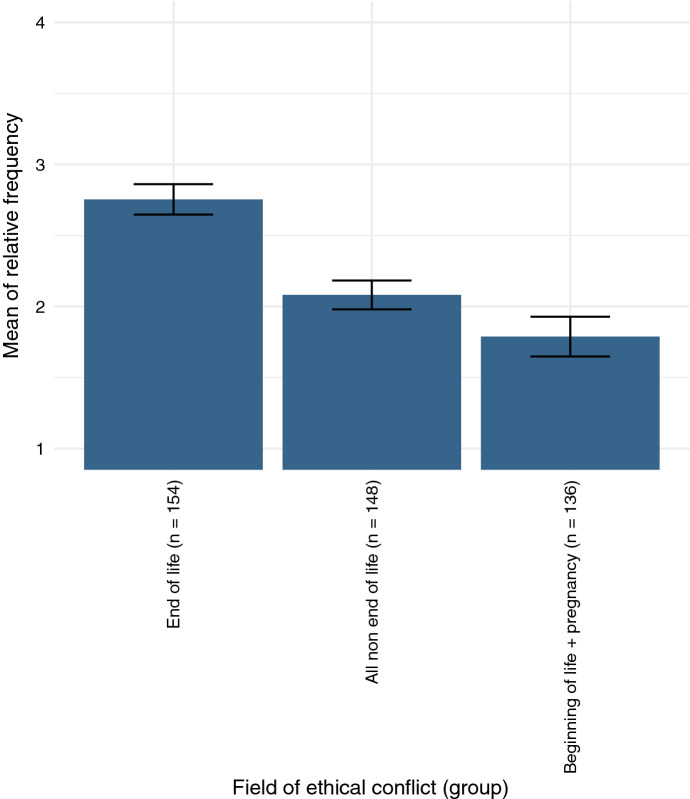
Comparison of different groups of ethical conflicts, displayed with 2 *σ* confidence intervals Group “All non-end of life” includes “beginning of life + pregnancy.” Scale: 1 = never, 2 = rarely, 3 = sometimes, 4 = often

When comparing end-of-life decisions with all other categories combined, end-of-life decisions appear to make up the largest portion of chaplains’ interactions with ethical decision processes (significant by 9.1 *σ*; cf. Fig. 2). For the purpose of this comparison, “limitation of therapy,” “advance care planning,” “suicide, suicidality, assisted suicide,” “palliative/terminal sedation,” “VSED (voluntary stopping of eating and drinking),” and “organ donation” have been counted as end-of-life decisions. The category “judgement in cases of dementia” could be debated, since it often is an irreversible condition leading to death. However, decisions on the power of judgement can occur in earlier stages of the illness process as well. It must be noted that these categories can only represent a subset of possible conflicts in the context of end-of-life decision-making.

On the other side of the spectrum, ethical conflicts surrounding the beginning of life as well as pregnancy occur less often overall. Figure 1 might, however, be slightly misleading in providing an insight into the general tendencies. Every category—even the overall lowest category “reproductive medicine” (1.4; between “never” and “rarely”)—had been rated as high as “often” by some chaplains, indicating that the types of conflict vary between different particular praxes, possibly being highly dependent on the units or specialized facilities at which chaplains worked.

Besides end-of-life decisions, chaplains indicated they were highly involved with conflicts surrounding culture and religions as well as organizational ethics.

Chaplains were also asked to provide information on further fields of conflicts, which occur at least “sometimes” in their praxis and have not been mentioned in the immediate categories. These categories were: administration of PEG tubes, high-risk surgery, visiting regulations during the COVID-19 pandemic, violence, and conflicts with/between relatives. Other conflict categories mentioned could be incorporated in the above-mentioned categories: for example, change of therapeutic target, feticide, and assisted suicide with EXIT (one of the assisted suicide organizations in Switzerland). However, the additional mentioning might indicate the significance of the particular category to the individual chaplain and their praxis.

#### Settings and Groups for Contact with Ethical Conflicts

**Fig. 3:**
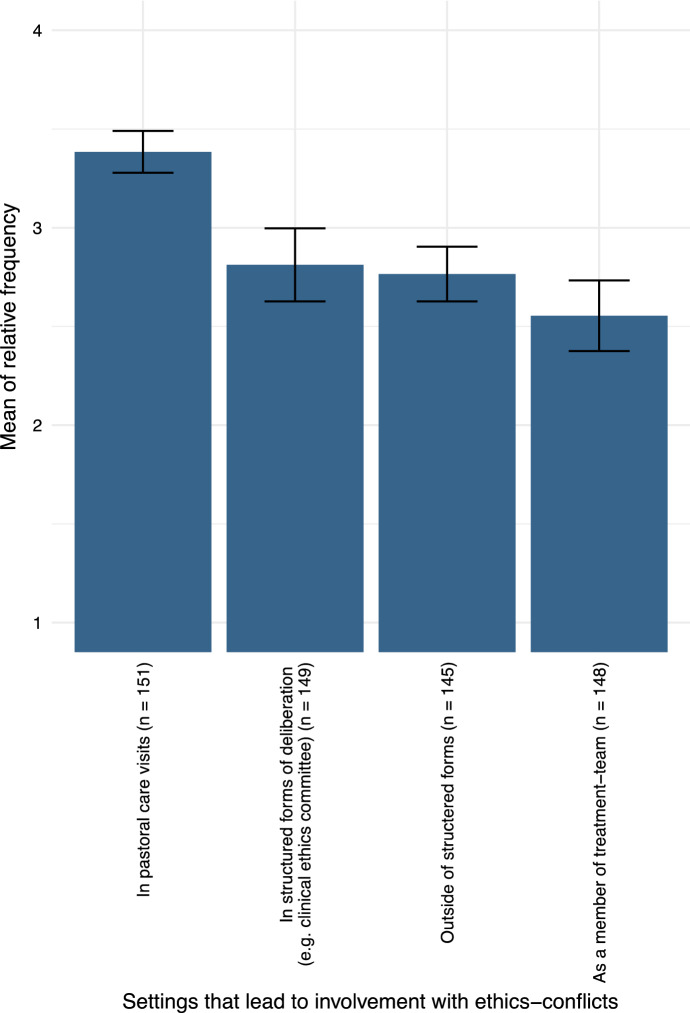
Communication settings that lead to chaplains being involved with ethics conflicts, displayed with 2 *σ* confidence intervals. Scale: 1 = never, 2 = rarely, 3 = sometimes, 4 = often

The chaplains responding to our survey agreed largely on getting in contact with ethical conflicts in their genuine praxis of “pastoral care visits” most often (mean 3.4, cf. Fig. 3). This finding is significant with 5.4 *σ* against the next highest category “in structured forms.” It appears to be inconclusive whether contact with ethical questions occurs more often in structured or non-structured forms of deliberation; however, both had a higher mean of relative frequency than being generally part of a treatment team.

**Fig. 4:**
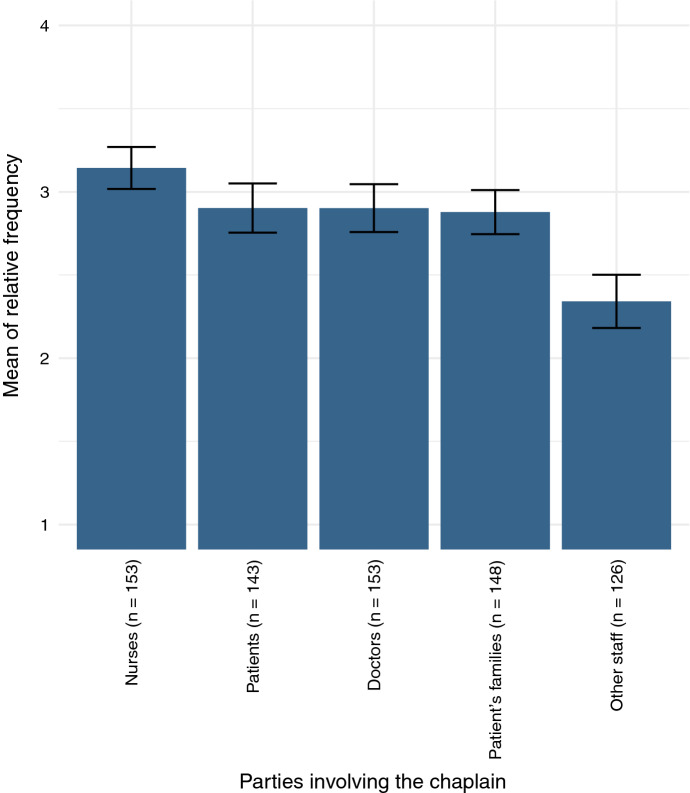
Party that involved the chaplain with the ethics conflict, displayed with 2 *σ* confidence intervals. Scale: 1 = never, 2 = rarely, 3 = sometimes, 4 = often

Chaplains interact with all different groups within the hospital—they can be pastoral caregivers for both patients and staff, but are approachable for ethical questions by all as well. Respondents in this survey indicated that “involvement with ethical conflicts” is most often established through nursing staff as shown in Fig. 4. This result is significant with over 2 *σ* against all other answer options, e.g., 2.5 *σ* tested against “Patient.” The differences between the middle three “Patient,” “Doctors,” and “Families” are inconclusive. Contact through other groups of staff is significantly less frequent.

#### Respondents Assessment of Chaplains’ Involvement

**Fig. 5:**
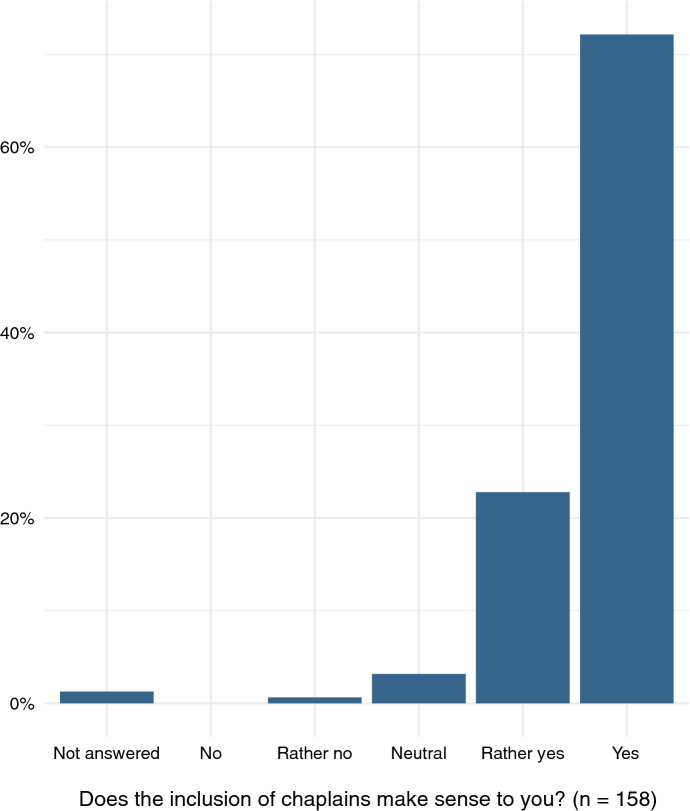
Responses to “Do you think it makes sense to include chaplains in ethics consultation?”

It must be noted that responding chaplains deemed chaplains’ involvement with ethics deliberation processes to be of high value. It has been the question with the highest value of agreement (72.2% responded with the highest possible score for this question; mean 4.7; cf. Fig. 5). Even though this deviates from their personal willingness to be involved (mean 2.8), a significant correlation between the two questions could be found (*r* 0.55, *r*^2^ 0.31, *p * ≈ 0).

#### Nonsignificant Results

There seems to be no significant correlation between higher levels of training in either pastoral care, spiritual care, ethics, or ethics counseling with an overall higher frequency of involvement in ethics deliberation. Only respondents with additional training in ethics counseling had a correlation with their basic willingness to be more involved with ethics processes; the effect size, however, is only minor (*r* = 0.18, *r*^2^ = 0.03, *p* = 0.027).

For this sample, no significant differences could be found between chaplains being involved with palliative and/or intensive care in relation to the ethical conflicts with which they were in contact.

In relation to the frequency overall for fields of ethics conflict, no significant difference could be detected for either sponsorship of position (church or hospital) or ownership of hospital. This survey can therefore provide no further insight into possible changes to the profession of chaplaincy due to changes in these categories or their specific position in healthcare institutions.

The rate of conflicts in the field of “suicide, suicidality, assisted suicide” did not differ significantly between Switzerland as a country with established organizations for assisted suicide and the others (German and Austria) where patients did only have scarce or no access to organized assisted suicide.

## Discussion

### Chaplaincy in End-of-Life Care

The comparison of areas of conflict surrounding end-of-life care with other types shows that the focus on end-of-life decisions in the former studies can be justified.

This suggests that ethical competence concerning end-of-life care issues is crucial for healthcare chaplains and should be supported by means of appropriate educational programs. Moos et al. (2016) noted before that chaplains overall felt quite unprepared to be faced with ethical conflicts in a professional manner. Due to our findings, we believe that “ethics at the end of life” should be specifically considered for further training with a high impact on chaplains’ praxis.

Chaplains could very likely be viewed as especially qualified and responsible in matters of death and dying by both patients and staff, since many of them are priests or pastors. This could lead to a bias of contacting chaplains in these situations and therefore chaplains being mostly confronted with questions surrounding end-of-life care. Additionally, due to usually short hospital stays of most patients, longer “visit series” predominantly occur with elderly patients in context of long phases of multimorbidity when other care facilities are not able to provide appropriate care anymore. However, it must be noted that all types of conflicts tested in this study were of high relevance to the practice of some chaplains.

### Chaplaincy and Assisted Suicide/Desire to Die

The data indicates that pastoral caregivers are remarkably often confronted with clients who have a desire to die or who consider assisted suicide. Nearly a quarter of respondents (23.4%) were faced with such questions “often” and another 40.5% “sometimes.”

As the questionnaire, though, did not further differentiate between wishes for assisted suicide and the desire to die, these findings need to be backed up by further and more in-depth research. The concept of a “desire to die” does cover a wide range of situations besides wishes for assisted suicide, like the desire for ending life-supporting treatment or a general feeling of being “old and full of days” like Job in the Bible (Job 42:17, KJV). Rehmann-Sutter et al. differentiate nine subtypes for this field of conflict ranging from “not considering hastening death” to “considering hastening death” as well as a general “acceptance of dying” (Rehmann-Sutter et al., 2017, p. 112). They also point out that there will always be a difference between the actual wish, its articulation, and the way it is understood. Thoughts of not wanting to live anymore are most likely present for many patients facing terminal illnesses during later phases of the illness and could therefore be present among the conflicts chaplains encounter in general (cf. e.g., Ehlert, 2014).

Since no significant difference in average frequency of occurrence of this particular field of conflict was found between countries with an established practice of assisted suicide (Switzerland) and the other countries represented in this study, no result on the impact of regulation can be reported.

### Structured and Non-Structured Forms of Ethics Consultation

While some earlier studies have researched the roles that chaplains take in structured forms of ethics consultation like clinical ethics committees (e.g., Anselm, 2008), Moos et al. (2016) were able to observe that a considerable amount of “contact with ethics questions” occurs in non-structured forms and especially within pastoral visits itself. Here, chaplains assisted both the process of making decisions as well as the process of dealing with decisions already made.

Our survey was able to back up these findings in a larger sample size: respondents associated “in pastoral care visits” with the highest frequency of all settings. It should be noted, however, that chaplains spend more time in pastoral counseling than working explicitly as members of teams, councils, or committees (e.g., a clinical ethics committee).

“Being part of the treatment team” had the lowest mean frequency; however, this is only significant tested against “in pastoral counseling”; tested against “outside of structured form of ethics consultancy” the result was not significant (1.9 *σ*). The test result for “within structured forms” and “outside structured forms” was inconclusive. It can therefore be assumed that both—structured and non-structured—forms of ethics consultancy are of importance to chaplains’ praxis of being involved with ethical counseling and decision-making.

Our findings also point to the fact that the ethical dimension of pastoral care visits itself needs to be evaluated by further research. Relevant questions could be what chaplains understand as ethical dimension or ethical conflicts within their pastoral care visits. This is closely related to the question of a professional ethics of pastoral care which would be contained in every aspect of their praxis. During this survey, it might have been easier for chaplains to point out ethical conflicts within their involvement of structured form of ethics deliberation like clinical ethics committees, because the role of “discussing ethics” is more obviously ascribed to these contexts.

### Settings and Team Involvement

Another focus of this survey has been to enlarge the data on communication settings and contact to other “groups in the hospital” that lead to chaplains being involved with ethical decision-making processes. This should contribute to a deeper understanding of the impact of chaplains’ position within the healthcare institutions.

As mentioned above (Fig. 4), in our findings nursing staff ranked the highest to involve chaplains in ethics processes. This could be in two ways: Either nurses directly mention ethical questions to the chaplains and ask them for support in dealing with them; or they refer chaplains to patient visits where they get in contact with ethical concerns (cf. Figure 3). Even though the latter option fits the data gathered well, both could be possible.

The effects of team involvement had been discussed in recent studies before. Often effects like higher involvement in ethics deliberation had been observed when chaplains were integrated in treatment teams or had been present at staff meetings (cf. e.g., Carey & Cohen, 2009; Carey et al., 2006, p. 26; Clemm et al., 2015, p. 47; Wirpsa et al., 2019, p. 30). This is closely connected to the ongoing discussion on the specific position of chaplains in Germany as simultaneously “insiders” and “outsiders” of the hospital system (cf. e.g., Bentele, 2010, p. 35; Janik, 2014, p. 301) which was also connected to the question of position funding. However, as mentioned above, “being part of a treatment team” was ranked the lowest among options to integrate chaplains in ethics deliberation in our survey. The data suggest that chaplains found other settings, especially pastoral care visits, to lead more often to being involved. Additionally, no significant correlations between funding and involvement in ethics processes were observed.

### Limitations

As we did not define a specific concept of ethics for the participants, they relied on their own understanding. We therefore must presume that they might have different understandings of what is an ethically relevant involvement. Accordingly, the results only show what the participants themselves understand to be practices of ethical deliberation or decision-making they are engaged in.

By proposing certain areas of conflict in medical decision-making as indicators for processes of *ethical* decision-making, the questionnaire implicitly suggests what we call a “conflict-based understanding of ethical decision-making”—according to which the role of ethics is to solve moral conflicts by means of normative deliberation. Such a conflict-based concept has been found to be prevalent in the context of clinical practice. It nonetheless does only cover one aspect of ethical questions as it excludes ethical deliberation about moral questions of a good life, which might be a crucial aspect of pastoral conversations with clients (Coors, 2015).

Nonetheless, as the research interest of the survey was the involvement of chaplains in clinical processes of ethical decision-making, this focus on a conflict-based understanding of ethics is appropriate. It must, however, be noted that the survey only covers one aspect of how chaplains are confronted with ethics and morality in the clinical setting.

## Conclusion and Outlook

Summing up, it must be noted that chaplains face a wide variety of fields of ethical conflicts in their praxes with an emphasis on questions surrounding the end of life. Contacts with such questions happen especially often in direct pastoral care visits or are referred through nursing staff. Due to the high relevance of these two fields to chaplains’ ethics deliberation praxes, they require more research effort in the future. The ethical dimension within interactions of immediate pastoral care (i.e., confession, prayer, ritual, and especially caring conversation) should be investigated deeply, for example by looking at different understandings of ethics among chaplains, an underlying professional ethics of caring for others, spirituality as an ethical concept, etc.

Overall, a deep connection can be assumed between pastoral care and ethics counselling—both come together in one professional person: the chaplain. Research—for example when utilizing qualitative interviews—should therefore focus on their specific role and unique contribution within this connection. This applies to both structured forms of ethics deliberation where chaplains are still pastors and non-structured forms or immediate pastoral care visits where chaplains are still experts for ethics.

## References

[CR1] Anselm, R. (Ed.) (2008). *Ethik als Kommunikation. Zur Praxis Klinischer Ethik-Komitees in theologischer Perspektive*. Universitätsverlag Göttingen.

[CR2] Bentele K (2010). Zur Rolle von Klinikseelsorgern in der klinischen Ethikberatung. Zeitschrift Für Medizinische Ethik.

[CR3] Carey LB (2012). Bioethical issues and health care chaplaincy in aotearoa New Zealand. Journal of Religion and Health.

[CR4] Carey LB, Cohen J (2008). Religion, spirituality and health care treatment decisions: The role of chaplains in the Australian clinical context. Journal of Health Care Chaplaincy.

[CR5] Carey LB, Cohen J (2009). Chaplain-physician consultancy: When chaplains and doctors meet in the clinical context. Journal of Religion and Health.

[CR6] Carey LB, Rumbold B, Newell C, Aroni R (2006). Bioethical issues and health care chaplaincy in Australia. Scottish Journal of Healthcare Chaplaincy.

[CR7] Clemm, S. (2015). *Die Rolle von Seelsorgerinnen und Seelsorgern bei Therapieentscheidungen und Ethikberatung am Lebensende* [Doctoral dissertation, Ludwig-Maximilians-Universität München] https://edoc.ub.uni-muenchen.de/18950/1/Clemm_Stephanie.pdf

[CR8] Clemm S, Jox RJ, Borasio GD, Roser T (2015). The role of chaplains in end-of-life decision making: Results of a pilot survey. Palliative & Supportive Care..

[CR9] Coors M (2015). Gesprächsräume als Urteilsräume: Der Beitrag der Seelsorge zur ethischen Urteilspraxis im Krankenhaus. Wege Zum Menschen.

[CR12] Deutscher Evangelischer Krankenhausverband e.V., Katholischer Krankenhausverb and Deutschlands e.V. (1997). *Ethik-Komitee im Krankenhaus*.

[CR10] Ehlert FS (2014). Wenn Menschen nicht mehr leben wollen: Sterbewünsche als ethische Herausforderung. Wege Zum Menschen.

[CR11] Janik, J. (2014). “Patients first” – Interreligiöse Klinikseelsorge als Qualitätskriterium in der Patientenbegleitung. In H. Haker, G. Wanderer, & K. Bentele (Eds.), *Religiöser Pluralismus in der Klinikseelsorge. Theoretische Grundlagen, interreligiöse Perspektiven, Praxisreflexionen* (pp. 297–310). LIT.

[CR15] King James Bible Online (2022). *The Holy Bible. King James Version.* https://www.kingjamesbibleonline.org

[CR13] Mandry, C., Sperneac-Wolfer, C., & Wanderer, G. (2019). *Klinikseelsorgerinnen und Klinikseelsorger als medizinethische Akteure. Profil und Kompetenzen. Ergebnisse einer partizipativen Interview-Studie*. https://www.uni-frankfurt.de/80984993.pdf

[CR14] Moos T, Ehm S, Kliesch F, Thiesbonenkamp-Maag J (2016). Ethik in der Klinikseelsorge.

[CR16] R Core Team (2022). *R: A Language and Environment for Statistical Computing.* https://www.r-project.org

[CR17] Rehmann-Sutter, C., Ohnsorge, K., & Gudat, H. (2017). Understanding terminally ill patients’ wishes to die. Significant narratives and a typology. In M. Bobbert, B. Hermann, & W. U. Eckart (Eds.), *Ethics and Oncology. New Issues of Therapy, Care, Research* (pp. 297–310). Verlag Karl Alber.

[CR18] RStudio Team (2020). *RStudio: Integrated Development Environment for R.* https://www.rstudio.com

[CR19] Vandenhoeck A (2021). The impact of the first wave of the covid-19 pandemic on chaplaincy in health care: Introduction to an international survey. Journal of Pastoral Care & Counseling.

[CR20] Wickham H (2016). gglot2: Elegant Graphics for Data Analysis.

[CR21] Wirpsa MJ, Johnson RE, Bieler J, Boyken L, Pugliese K, Rosencrans E, Murphy P (2019). Interprofessional models for shared decision making: the role of the health care chaplain. Journal of Health Care Chaplaincy.

